# Mr. Hoff and the New York Academy of Medicine

**Published:** 1867-08

**Authors:** 


### Mr. Hoff and the New York Academy of Medicine.

At the last meeting of the Academy of Medicine, the following resolutions were unanimously adopted:

*Whereas*, W. L. Hoff, proprietor or agent of the "Hoff Malt Extract," is issuing publications through the secular papers, and by means of pamphlets and circulars professing to quote favorable opinions expressed in a report of a committee of the Academy;

And, *Whereas*, the said Hoff is widely circulating a letter purporting to have been written by a Fellow of the Academy;

And, *Whereas*, the publications of said Hoff are so adroitly and designedly worded as to impress the mind of the reader with the belief that the Academy has endorsed his nostrum, and has thus apparently compromised its dignity and professional standing; therefore,

*Resolved*, That the New York Academy of Medicine does hereby proclaim and declare that it has not expressed any opinion in regard to "Hoff's Malt Extract," and that any and every use of its name in recommending said Extract is unauthorized by the Academy.

*Resolved*, That a copy of the above preamble and resolutions be sent to the medical journals of this city, and that the medical journals throughout the country be requested to copy the same, in justice to the Academy and the profession.

